# A novel muscle-friendly technique with 90° dissection plane rotation in tunneling endoscopic submucosal dissection

**DOI:** 10.1055/a-2641-2128

**Published:** 2025-07-25

**Authors:** Ahmad Madkour, Ahmad F. Aboelezz, Hassan Atalla, Osama Elnahas, Alaa Ismail, Hossam Ismail, Amr Elfouly

**Affiliations:** 1 575928 Endemic Medicine Department, Helwan University, Faculty of Medicine, Cairo, Egypt; 2 68781 Department of Internal Medicine, Gastroenterology and Endoscopy Unit, Tanta University, Tanta, Egypt; 3 68780 Department of Internal Medicine, Hepatology and Gastroenterology Unit, Mansoura University, Mansoura, Egypt

Endoscopic submucosal dissection (ESD) is a minimally invasive, organ-preserving maneuver that can be used to resect early gastrointestinal tumors, even larger ones. In recent years, different ESD strategies have been applied to facilitate resection of large lesions, like the tunneling technique and the pocket creation method 1 2 3 4 .

Despite the advantages provided through tunneling ESD, it carries technical challenges such as the narrow working space, looping of the scope inside the tunnel, and false orientation of the scope, resulting in injury to the muscle or the inner surface of the mucosal flap.

Here, we report the 90° dissection plane rotation via the scope, where the muscle bed and the mucosa became on the right and left sides, respectively, instead of the upper and lower planes. In this technique, a wide incision from the cecal side of the lesion through retroflexion of the scope was done at first. This is followed by another incision at the anal side where we start entering our tunnel ( Fig. 1 ). This novel muscle-friendly approach provides clear visualization of the dissection plane and good orientation of the scope throughout the procedure. Moreover, this helps in proper assessment and dealing with the penetrating vessels. To address, this technique was found to be beneficial in sigmoid colon lesions where the scope position is unstable, and the colon wall is redundant and floppy ( Video 1 ).

**Fig. 1:**
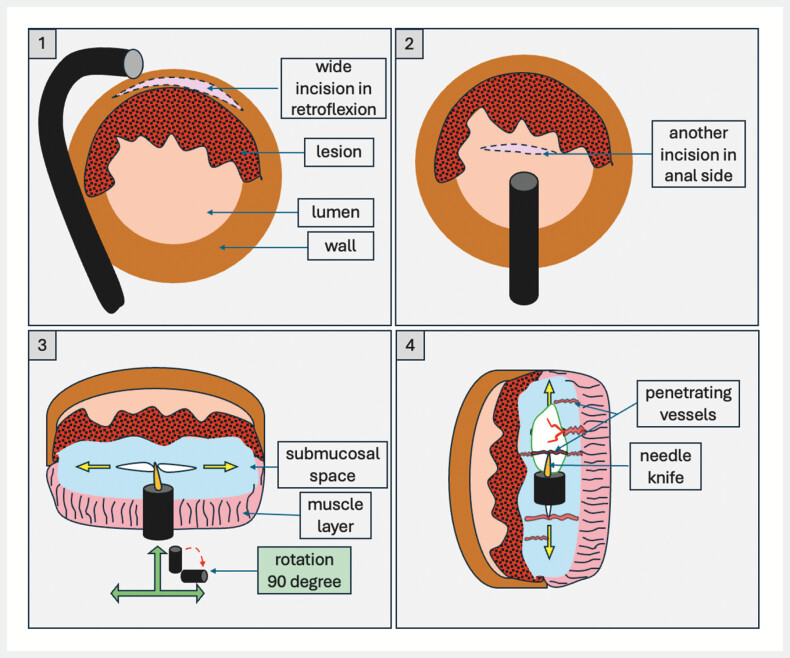
Schematic representation of the endoscopic view after 90° rotation with the muscularis propria and mucosal flap on both sides of the dissection plane.

The video demonstrates a novel muscle-friendly technique with 90° dissection plane rotation in tunneling ESD.Video 1

**Patient 1** A 44-year-old female patient with a lateral spreading tumor, granular mixed type. Histological assessment confirmed R0 resection of a tubulovillous adenoma with high-grade dysplasia.

**Patient 2** A 72-year-old man presented with a large circumferential anorectal lesion measuring about 32 cm. En-bloc resection was achieved, and histopathological examination revealed intramucosal carcinoma.

**Patient 3** A 58-year-old female patient with a large sigmoid lesion, and histopathological examination revealed an intramucosal carcinoma that was removed en bloc.

Endoscopy_UCTN_Code_TTT_1AQ_2AD_3AZ

## Notes

Endoscopy E-Videos https://eref.thieme.de/e-videos
*E-Videos* is an open access online section of the journal *Endoscopy* , reporting on interesting cases and new techniques in gastroenterological endoscopy. All papers include a high-quality video and are published with a Creative Commons CC-BY license. Endoscopy E-Videos qualify for HINARI discounts and waivers and eligibility is automatically checked during the submission process. We grant 100% waivers to articles whose corresponding authors are based in Group A countries and 50% waivers to those who are based in Group B countries as classified by Research4Life (see: https://www.research4life.org/access/eligibility/ ). This section has its own submission website at https://mc.manuscriptcentral.com/e-videos .
